# A framework for siting public EV charging during renewable-aligned hours

**DOI:** 10.1016/j.isci.2026.117198

**Published:** 2026-08-13

**Authors:** Nicolas Williams, Salomon A. Mendoza, Esteban A. Lopez Ochoa, Kristen E. Brown

**Affiliations:** 1 Department of Biomedical Engineering & Chemical Engineering, University of Texas San Antonio, San Antonio, TX, USA; 2 School of Architecture + Planning, University of Texas San Antonio, San Antonio, TX, USA; 3 School of Civil & Environmental Engineering, and Construction Management, University of Texas San Antonio, San Antonio, TX, USA

**Keywords:** EV charging stations, duck curve, demand-side management, electric vehicles, EVs

## Abstract

Utilities must incorporate electric vehicle (EV) charging demand alongside variable generation. Early EV adopters relied on home charging, creating evening loads poorly aligned with solar. Existing demand-side management strategies often rely on time-of-use pricing or automated control, which can limit equitable access. As public charging is deployed, location selection may support charging when solar is abundant. This study develops a replicable, data-driven framework to identify candidate locations for public EV charging based on the overlap between observed visitation and grid-favorable charging hours. The preferred time period for charging in Texas is from 10 a.m. to 3 p.m. Using mobile phone location data for a case study in San Antonio, the framework identified over 100 candidate locations with visitation patterns aligned with this window. Promising locations include retail centers, financial institutions, and lunch-oriented restaurants. Rather than optimizing charger operation after deployment, the framework provides a siting approach to align public charging with demand-side management goals.

## Highlights

• Siting public EV charging is reframed as a demand-side management strategy
• Analysis identifies potential sites popular during solar-aligned hours
• Visitation-derived charging is compared to observed public charging behavior

## Graphical abstract

**Figure:**
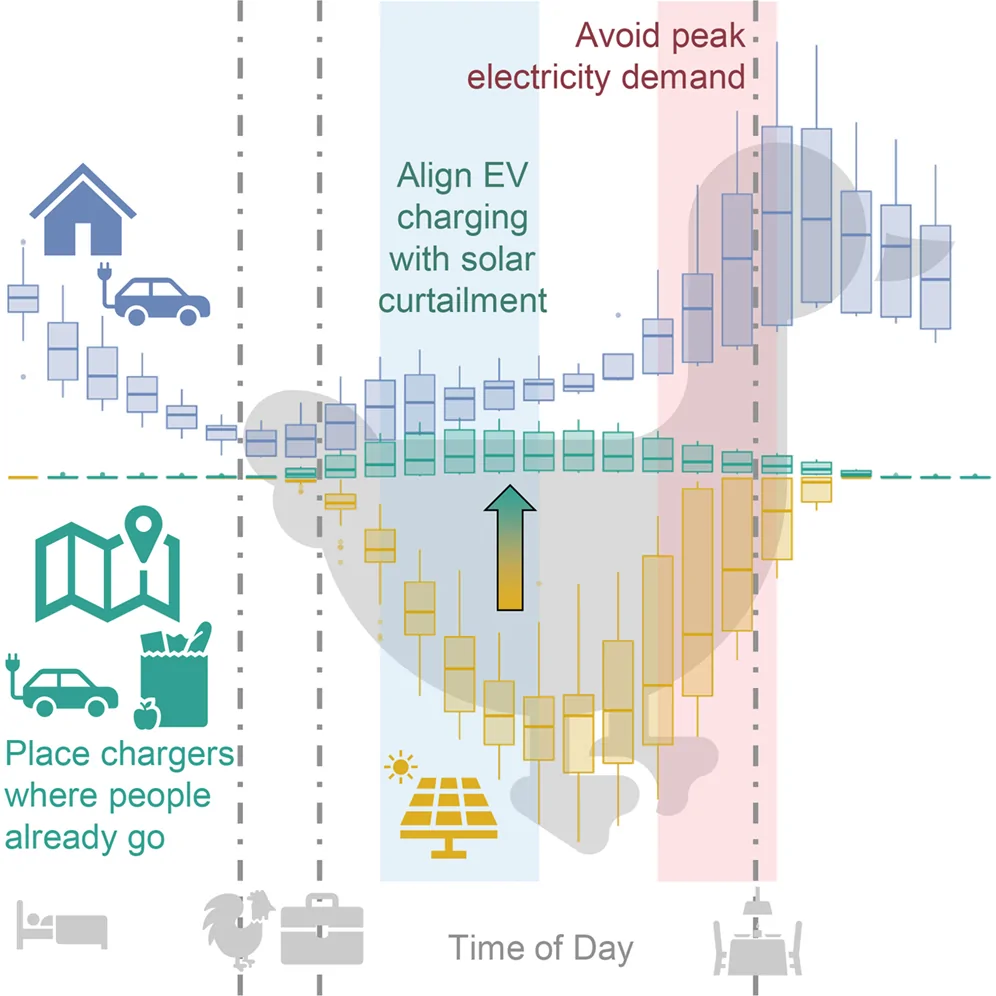

## Teaser

Applied sciences; Computer science

## Introduction

Electricity systems are increasingly using a wider mix of energy sources for generation while facing a more complex portfolio of demands. The changing loads include the electrification of heating and transportation and the emergence of advanced digital loads at both the industrial and consumer scale. The increased contribution of variable renewable energy (VRE) sources over the same time period adds further complexity. One engineering challenge intensified by this new paradigm is that of matching electricity supply and demand with precise temporal accuracy.

Traditionally, transportation favored liquid fuels, which could be refined and stored until needed. Conversely, the electric power grid requires that the generation and consumption of electricity are always balanced in real time. Traditional power grids were designed around large, centralized fossil-fuel generators that vary generation to meet fluctuating demand. One of the most distinct representations of these growing challenges is the “duck curve,” which describes the mismatch between afternoon-peaking solar generation and a typical evening peak in electricity demand.^1^

The duck curve describes how net load changes throughout the day as more solar power is integrated into the electrical grid. The curve gets its name because, when plotted, it resembles the shape of a duck, with a low belly transitioning to a neck and head. Three primary concerns associated with the duck curve phenomenon are underutilization of renewable generation, steep ramping requirements, and grid instability associated with increased wear-and-tear on grid infrastructure.^2^ In power systems, curtailment refers to the process of restricting energy production, particularly from VRE sources like solar and wind, to avoid overloading the grid. Ramping—the process of rapidly increasing or decreasing power output—is essential during changing loads, but this drastic transition strains assets. In addition, quickly ramping up electricity production often requires the use of generation technology with higher emission rates.

Baseload demand, the power that is consistently required at all times of day, is typically met by relatively inflexible generators such as those using nuclear energy or natural gas combined cycle, while intermediate and peak demands are served by more flexible generators such as gas turbines.^3^ Solar and wind outputs are dependent on natural cycles rather than grid needs. Thus, the energy control paradigm must expand beyond supply-side flexibility. As solar development increases, surplus solar generation could be managed through EV charging.^4^ In summer, cooling accounts for more than 50% of demand and over 70% of peak demand.^5^ While in winter, the solar peak is shorter, and demand in the study region is also lower due to decreased cooling load.^4^

Demand-side management programs influence consumers to shift or reduce electricity consumption during periods of high demand. When successfully implemented, these programs can reduce the strain of peak demand, decrease renewable energy curtailment that occurs during periods of low demand, and lower stress on the grid. The purpose is to shift flexible loads such as washing machines, electric vehicle (EV) charging, and space cooling away from peak periods to off-peak times.

Demand-side management strategies can also unintentionally heighten the energy burden, defined as the percentage of income spent on energy bills, for lower income or otherwise marginalized households. Under one common strategy, time-of-use pricing, consumers pay more for electricity during peak times.^6^ If a household cannot readily shift energy usage, invest in new appliances, or adapt to automated systems, it risks higher costs that reinforce inequities. Under the principle of energy justice, there should be equitable access to affordable, reliable, and clean energy,^7^^,^^8^ including public EV charging.^9^

Another demand-side management approach uses Internet of Things (IoT) devices, such as smart thermostats or network-connected appliances to automate demand response.^10^ These systems are designed to respond to real-time signals from the grid or utility to regulate power consumption, automating load shifting. Equity concerns also arise with this technique, as households without stable internet access or the financial capacity to invest in IoT devices may be excluded. Consumer acceptance and comfort also pose barriers, as households may only be willing to shift energy usage until it disrupts their daily routines.^7^

EV charging offers a unique opportunity for demand-side management because EVs already have batteries and can provide energy storage.^11^ Further, charging behavior is not associated with deeply entrenched patterns and may be more malleable. As EV costs decrease, more charging demand is expected.^12^^,^^13^ Many efforts to optimize EV charging have been reported^14^^,^^15^^,^^16^^,^^17^; however, most current strategies to shift EV charging utilize time-of-use pricing and IoT devices,^18^^,^^19^ which are still limited by energy justice concerns. These approaches are valuable for operational control, but they often assume that infrastructure already exists. As a result, they provide less guidance for upstream public infrastructure planning. In addition, they do not consider the opportunity to address demand-side management at earlier planning stages. Further, EV adoption itself is limited by charger access.^20^^,^^21^ This study addresses that gap by identifying candidate public charging locations before deployment using observed visitation patterns and region-specific timing.

By incentivizing or scheduling charging to coincide with times of excess renewable generation or low net load, EVs can effectively serve as a controllable demand resource. Despite growing interest in EV-based demand-side management, limited work has explored the use of public charging infrastructure itself as a mechanism to support demand-side management. To address this gap, we propose identifying locations for public EV charging stations based on occupancy during hours when additional electricity demand would be easier for the grid to accommodate.

**Figure 1:**
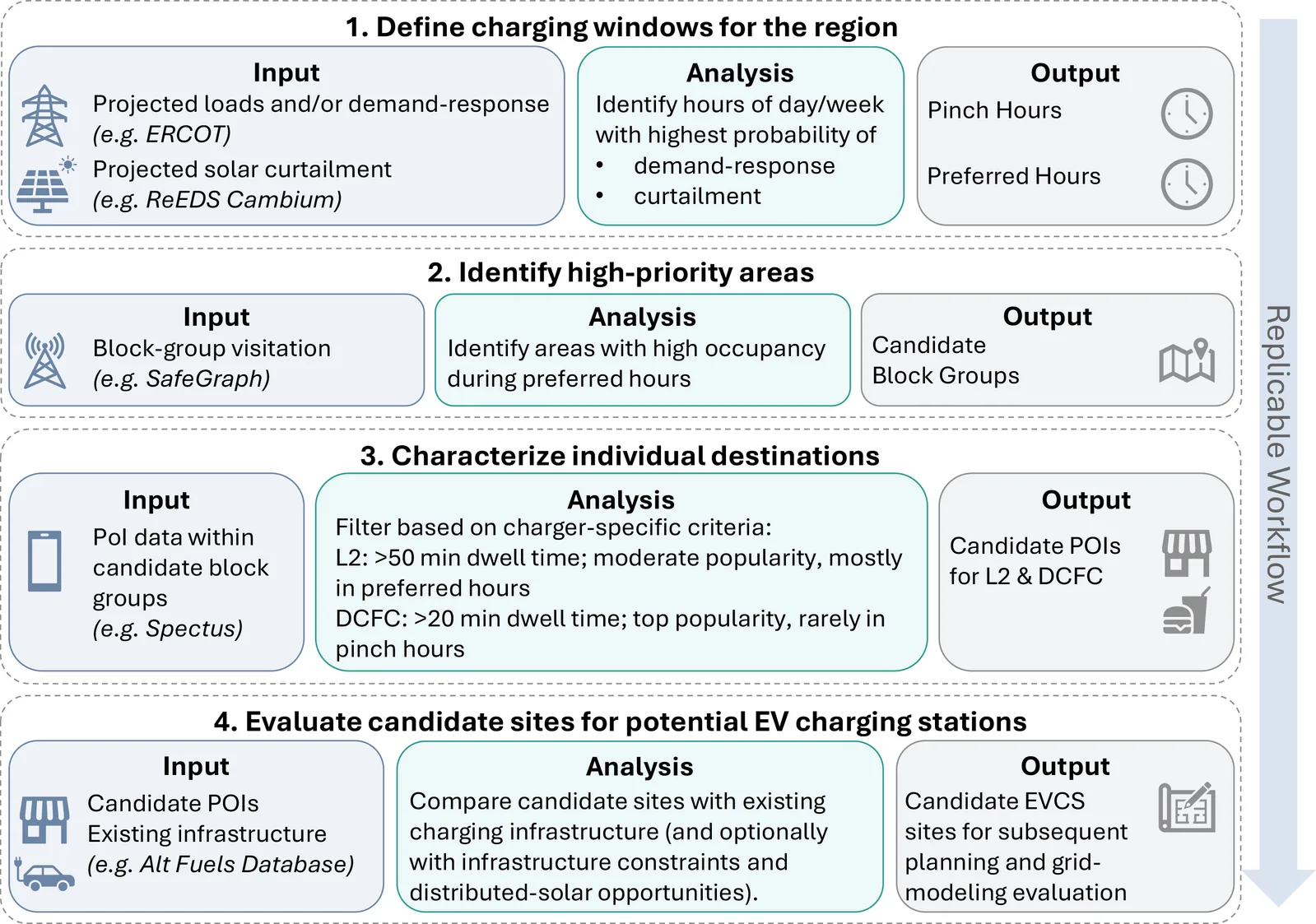
Workflow for replicating EVCS site identification The generalized steps to identify candidate sites for electric vehicle charging stations (EVCSs) are shown as a replicable sequence. For the inputs to each step, the data source used here is presented in italics, but other data providers may be more relevant based on the specific context. The output from each step is used in the next step. For this study, projected grid conditions for Electric Reliability Council of Texas (ERCOT) are used to identify “pinch” hours, when the grid is already stressed, and “preferred” hours, when additional EV charging would be easiest to incorporate. These time windows inform mobility-based filtering of points of interest (POIs) to identify candidate locations for public charging infrastructure.

This study presents a data-driven planning framework to identify locations for public EV charging infrastructure that aligns typical bulk grid conditions with observed travel behavior. Prior EV charging studies have often emphasized time-of-use pricing,^17^^,^^18^^,^^19^ techno-economic analysis,^22^^,^^23^^,^^24^ or mathematical optimization of charging operations.^11^^,^^15^^,^^25^ These approaches, while valuable, do not always address city-scale planning decisions, accessibility issues, or driver behavior. The framework presented here addresses these gaps by identifying candidate charging locations as points of interest (POIs) that align with identified grid needs and consumer behavior. The resulting candidate sites are not presented as globally optimal or grid-validated locations; rather, they provide a reproducible, data-driven basis for infrastructure planning and subsequent grid-modeling studies. Because the framework relies on commonly available grid projections and mobility data, it can be applied at the municipal level and adapted to region-specific constraints. While the analysis is presented for San Antonio, Texas, the underlying methodology is generalizable to other regions. An overview of the study design and data workflow is presented in Figure 1.

## Results

The Electric Reliability Council of Texas (ERCOT) manages the flow of electrical power to over 26 million customers and represents around 90% of Texas’ electrical load. Natural gas remains the dominant energy source for electricity generation in Texas,^26^ but the state also leads the nation in wind energy capacity, and total installed solar has been expanding rapidly.^27^ ERCOT currently has over 30 GW of operational solar capacity, although it is primarily utility scale, and distributed (rooftop) solar capacity is smaller than that of other states with high solar capacity.^28^ While capacity represents the theoretical maximum output, actual generation varies. Wind generation tends to be strongest during nighttime and early morning hours, while solar production peaks in the early afternoon. In contrast, electricity demand is typically highest in the late afternoon and early evening. This temporal mismatch motivates demand-side management.

**Figure 2:**
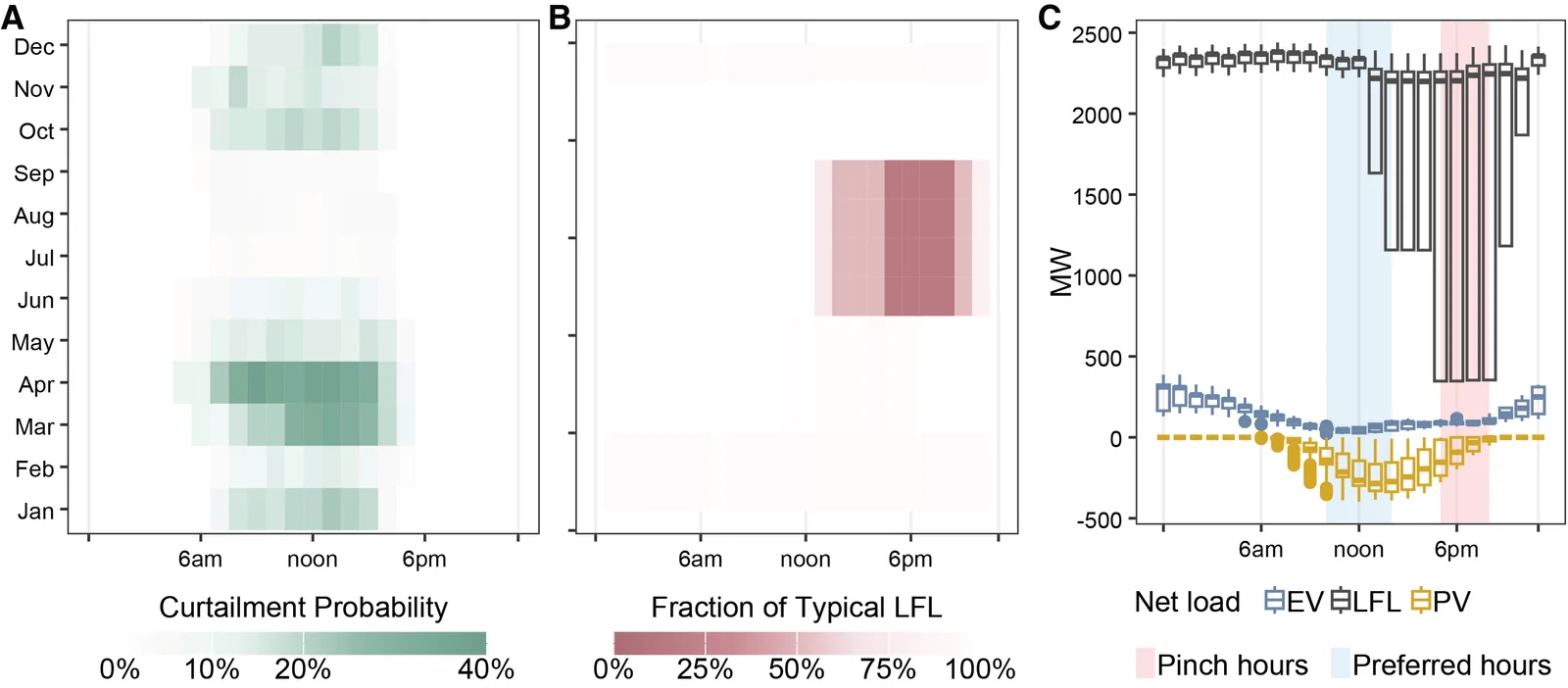
Time selection for South Central Texas (A) Probability that solar generation would be curtailed in South Central Texas in a given hour for each month. (B) Indication that demand response would be invoked for large flexible loads (LFLs) represented as the fraction of the typical (median) load. Values over 100% are shown as 100%. (C) Projected loads by time of the day, showing the LFL typically representing consistent demand aside from the demand response times, electric vehicle (EV) charging loads, and the load reduction from distributed (rooftop) solar photovoltaic (PV) generation. The pinch times indicate hours in which EV charging should be minimized based on the projected desire for demand response, and the preferred time indicates the time period that would be most beneficial for additional load as it aligns with periods of likely curtailment. Boxes indicate the interquartile range (25th–75th percentiles), center lines indicate the median, whiskers extend to the most extreme values within 1.5 times the interquartile range, and points beyond the whiskers indicate outliers.

This project evaluates a sub-region of ERCOT covering South Central Texas, which includes the Austin and San Antonio metro areas. Currently, the Austin area has about twice as many EVs as San Antonio,^29^ but the analysis here focuses on adding charging station locations only to San Antonio. Figure 2C includes EV and photovoltaic (PV) loads for the region. ERCOT planning documents^30^ indicate that small (<1 MW) solar PV installations, typically residential rooftop solar, and EV charging are two points of significant change in the grid for the next decade. By 2029, EVs are projected to need 6.7 TWh of annual energy consumption,^31^ and demand from light-duty vehicles (LDVs) is expected to peak between 7 and 8 p.m. on weekdays due to the use of at-home, level 2 chargers.

### Preferred charging times

Public charging may help alleviate curtailment by increasing demand during high solar generation, reducing the gap between the generation and demand. Preferred charging periods were defined as hours with the highest likelihood of solar curtailment, which is when strategic demand shifts could improve renewable energy utilization.

Projected curtailment is plotted in Figure 2A. We have identified the target charging window as between 10 a.m. and 3 p.m., corresponding to hourly time bins beginning at 10 a.m. through 2 p.m. Based on the projected curtailment in 2030, 2035, and 2040,^32^ we identified the hours of the day with the highest probabilities of solar curtailment (top 20%). This resulted in a threshold of 13% probability of curtailment in that hour across all months and years. We refer to this as the “preferred” time and highlight it in bluish-green in figures throughout the paper.

We also identified the time period from 5 to 8 p.m. as the “pinch” time because this is when demand may already stress the grid. This pinch time was identified using projected demand response requests for large flexible loads (LFLs) in the region (Figure 2B), indicating that the grid operators expect the grid to be stressed. This time period, highlighted in rose in figures, is when we are attempting to reduce charging.

### Candidate charging locations

Locations well suited for charging are those with high occupancy during the preferred time and low potential utilization during the pinch time. POIs are determined by the number of visitors in an hour and average dwell time. Well-situated POIs can enhance convenience and efficiency by aligning charging with regular routines to avoid idle waits or standalone charging. Different filtering algorithms were used to allocate level 2 and direct current fast charging (DCFC) sites. Locations with dwell times of at least 50 min are preferred for level 2 chargers (see Table 1), while shorter dwell times may work for DCFC as fast chargers can completely charge a car in as little as 20 min.

**Table 1:** Charging times assuming a 60-kWh battery installed within a light-duty battery electric vehicle^33^

Charger type	BEV charge time from empty (hours)	Electric range per hour of charging (miles)	Supply voltage (V)	Typical location
Level 1	40–50	2–5	120	home
Level 2	4–10	10–20	240	home, workplace, public
Level 3 (DCFC)	0.3–1	180–240	480	public

Charger types range from level 1 “trickle” chargers typically used at home to direct current fast charging (DCFC) devices found at public locations.

The criteria for level 2 sites are that the average dwell time should be at least 50 min, and overall popularity must not be in the bottom third of considered sites. In addition, average popularity of the site in the preferred hours should be at least half of the peak count, average popularity in pinch hours should be less than half of the peak, and popularity in any summer pinch hour should never exceed 60% of the peak. This resulted in the identification of 117 level 2 POIs.

As DCFC increases the load much more, stricter criteria are used to avoid fast charger use during the pinch times. As with level 2, average visitation in preferred hours must be at least 50% of the peak popularity, but average visitation in the pinch hours must be in the bottom quarter. No summer pinch hour could have visitation greater than half the peak value. DCFC sites require average dwell time to be at least 20 min, but total popularity must be in the top quarter to justify the investment. Seventeen POIs were identified for DCFC.

**Figure 3:**
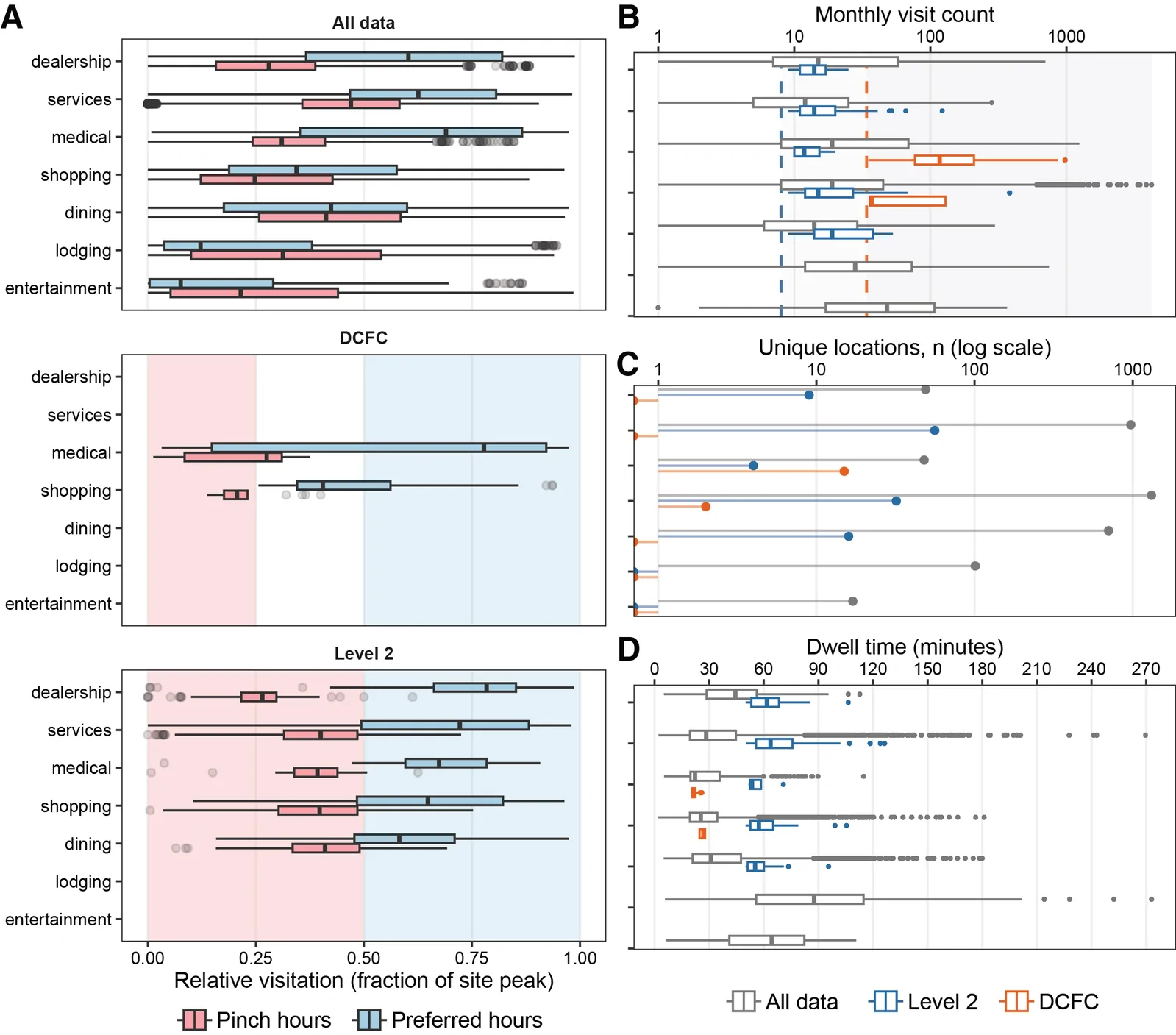
Comparison of selected points of interest against all candidate locations “All data” includes all POIs evaluated, “level 2” includes only sites identified as appropriate for level 2 charging, and “DCFC” includes only sites identified as appropriate for direct current fast charging (DCFC). POIs categorized as “vehicle related” are excluded from the figure, and category labels are consistent across plots but are shown only in (A). Boxes indicate the interquartile range (25th–75th percentiles), center lines indicate the median, whiskers extend to the most extreme values within 1.5 times the interquartile range, and points beyond the whiskers indicate outliers. (A) Relative visitation by category, normalized to peak occupancy of each POI, during the pinch and preferred time periods is shown, with background shading indicating filtering thresholds applied in the site selection process. (B) The number of monthly visitors in the dataset at each POI, with dashed lines indicating the minimum thresholds used for filtering. (C) The number of unique POIs for each category in each dataset. (D) The average dwell time per visit at POIs within the category.

Locations were identified independently but are grouped by category for reporting. Additional filtering removed “vehicle-related” locations such as car washes and vehicle repair facilities because charging may not be compatible with the existing activities. The “all data” comparison group in Figure 3 includes only POIs in the census block groups identified during the SafeGraph-based filtering. The effect of filtering and the typical characteristics of the identified sites are presented in Figure 3.

The locations identified for new fast charging stations include warehouse membership clubs and medical facilities that have high rates of visitation but short dwell times. The locations identified for new level 2 chargers include retail shopping locations, various services, dining locations, and car dealerships. Given the minimum dwell time requirement, several service-based institutions where people may spend approximately an hour appear on this list, including 17 mailing and shipping locations, 11 salons, 10 financial institutions, 7 cellular retailers, and 5 insurance agencies. Sixteen dining locations were identified, primarily fast-food restaurants or those located in areas near other businesses where people can stop in quickly for lunch during the workday or while completing other errands, such as sandwich or coffee shops. Another popular destination type is shopping venues, including a pharmacy, a mall, and grocery stores. The recommended locations are mapped in Figure 5.

### Current EV charging paradigms

EV charging behavior can influence supply-demand imbalances. Each charging paradigm exhibits distinct temporal patterns. Most common for early adopters is home charging, in which EV owners with private residences tend to plug in their vehicles after arriving home from work at around 6 p.m. This charging paradigm can add strain at peak times on the grid. Workplace charging, which often begins in the morning, does not align with solar generation and can coincide with wintertime heating demand. Other common paradigms are fleet and destination charging. The charger level can influence how successfully charging can be assimilated into user routines and grid load.^34^ We present a framework to encourage demand-side management through placement of destination charging.

We compared the suggested electric vehicle charging station (EVCS) locations to the existing public charging locations in San Antonio as reported in the DOE Alternative Fuel Station database.^35^ We limited the statistics to stations that are not only listed as public in the database but also filtered out those that are not broadly accessible. This includes removing those that are only for customers, employees, or guests and those in apartment complexes. There are currently 203 level 2 and 52 DCFC public charging stations. Based on the existing stations, the median level 2 EVCS has 6.5 kW power output with 2 ports per station, although some stations have up to 12 ports. The median DCFC station provides a power output of 150 kW with 4 ports per station, and the highest number of ports at a DCFC station is 8.

### Projected charging profile

**Figure 4:**
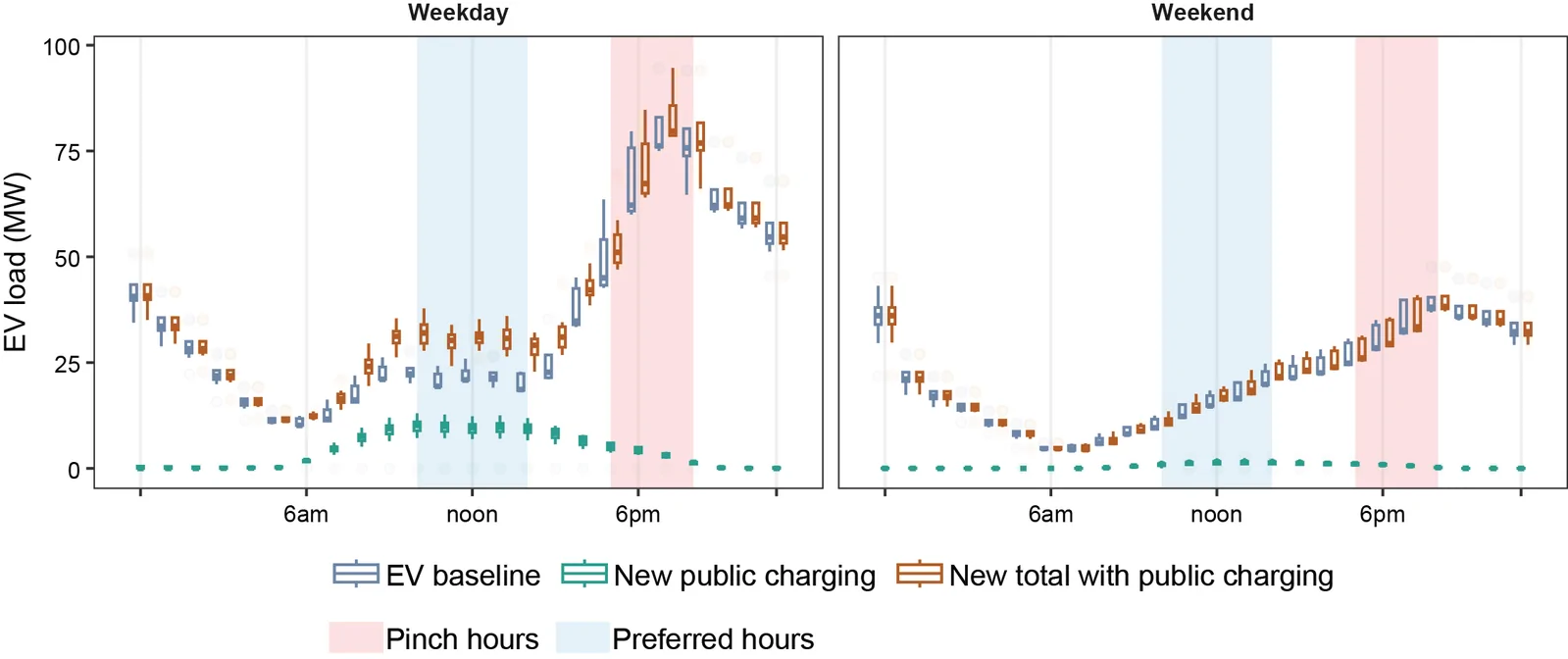
Charging profiles The original projected charging profile for 2025 in the South Central region of ERCOT (covering Austin and San Antonio, TX) is compared against the charging profile assumed for the new public EVCSs and a new composite charging profile. Variation within each hour is due to differences in behavior across days of the year. Boxes indicate the interquartile range (25th–75th percentiles), center lines indicate the median, whiskers extend to the most extreme values within 1.5 times the interquartile range, and points beyond the whiskers indicate outliers.

Assuming that installed EVCSs would be used in proportion to the current visitation rate for a location, a new projected EV charging load profile was constructed. Based on the filtering criteria, 17 locations were identified for fast chargers, and 117 locations were identified for level 2 stations. Charging demand was assumed to scale with relative visitation at a site, with maximum charging (13 kW for level 2 or 1 MW for DCFC) during the hour when the site is most heavily visited. Given these assumptions, the charging load added at one time across all new stations is projected to be at most 13 MW, approximately 70% of the theoretical maximum if all selected sites were fully utilized at once. The prior and new projected EV charging load for South Central Texas is plotted in Figure 4. The variation in demand shown using boxplots is associated with varying behavior during different days of the week and months of the year.

The daily total demand from the new public charging at the suggested sites under our assumptions is between 8.3 and 128.4 MWh. Of this amount, 4.4–62.5 MWh of additional charging occurred during preferred hours.

## Discussion

In this analysis, new public charging is still a modest contributor (<10%) to total EV charging. This is partly due to a preference for home charging and limitations on infrastructure buildout and partly due to the current scope considering adding charging stations only in San Antonio. However, even a modest contribution could affect grid conditions. Figure 4 shows the EV charging demand expected in South Central Texas from ERCOT projections and the new public charging demand based on our assumptions. The new overall charging profile does not reduce the total charging demand, as range anxiety may lead people to charge in public as well as at home. However, if midday public charging displaces evening charging, there could be additional benefits.

### Existing EVCSs

**Figure 5:**
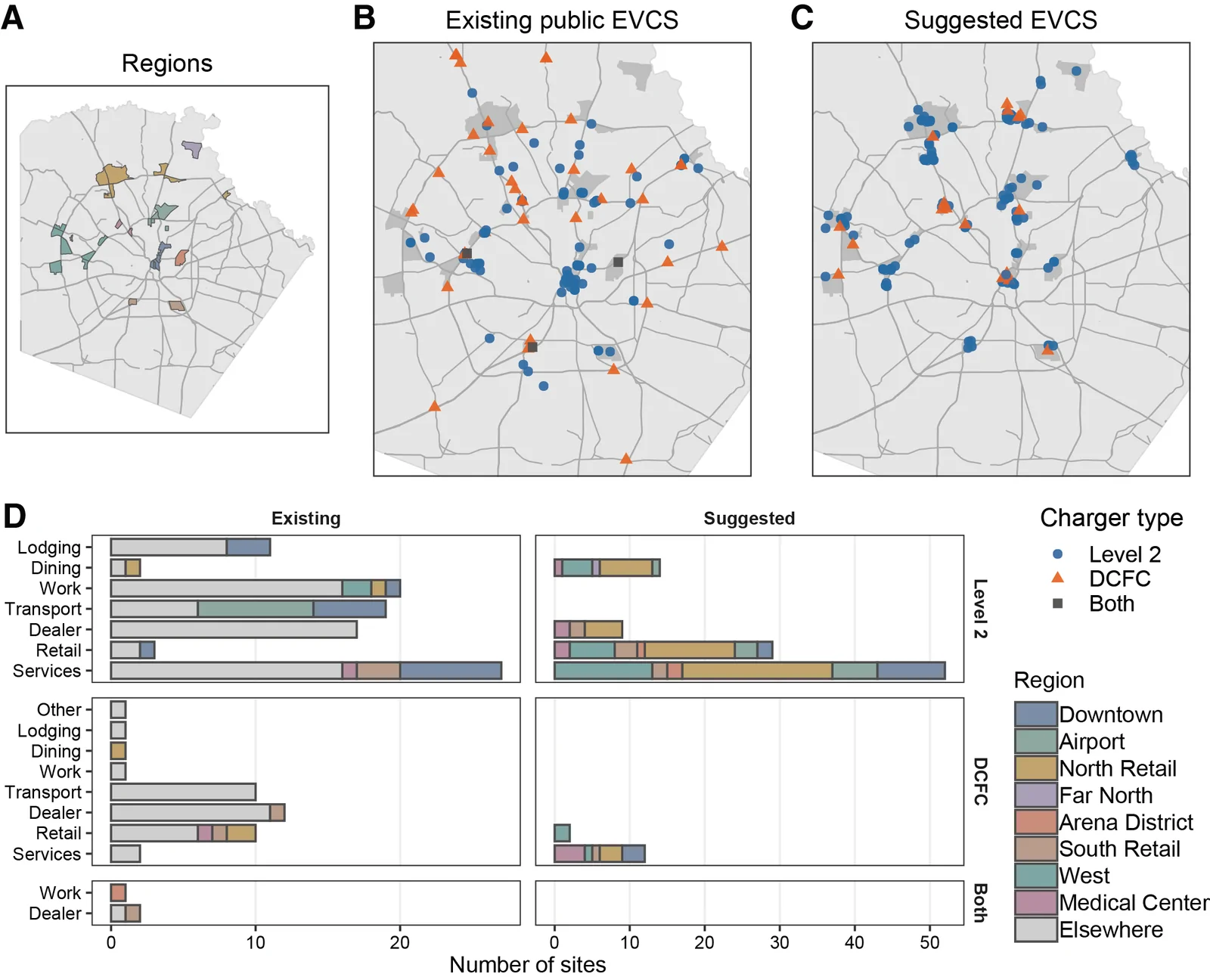
Existing and suggested charging locations (A) General regions of the city used to categorize locations. (B) Currently existing public electric vehicle charging stations (EVCSs). (C) Suggested EVCS to be installed based on the analysis here. (D) All existing and suggested sites were categorized in two ways: the type of operation or business category associated with the POI, and the general regions of the city shown in (A). Existing charging stations that are not near any of the suggested locations are just identified as “elsewhere.”

Many EVCSs have already been installed in San Antonio, and there are many reasons for their location choices. Therefore, the current locations are not necessarily aligned with the suggestions presented here. The proposed and current EVCSs by category can be compared in Figure 5. Of the public EVCSs that currently exist in the study area, many are at the same types of businesses. Categories of POIs present in the existing EVCSs but not identified in those recommended here include hotels and workplace, which are common charging locations but do not necessarily improve utilization of renewable energy.

Many existing stations are located at transportation-oriented sites such as fueling stations and parking facilities. Fueling stations, which typically do not have long dwell times, align with the traditional liquid fueling paradigm rather than the convenience paradigm sought here. Only one existing EVCS is at a vehicle repair location. We have intentionally excluded such vehicle-related locations from our suggestion, as we believe this is a category where assuming typical visitation aligns with future EV drivers is unlikely to be true.

In addition to the differences in categories served by the existing EVCSs, existing and proposed networks also differ spatially. Highly trafficked block groups were aggregated into several regions of the city for comparison, identified on the map in Figure 5A. While some of the identified block groups are well covered by existing EVCSs, those further from the city center tend not to be. The downtown area already has more EVCSs than suggested by the framework. Strikingly, the “north retail” area, composed of several large mixed-use commercial developments, appears to be significantly underserved by existing infrastructure. The western portion of the city is also underrepresented in the existing sites, although there are several charging stations just outside the identified boundaries. This analysis of the existing infrastructure indicates that prior planning was guided by different objectives.

### Scale of the solution

Projected EV charging demand in the near future will remain relatively small compared to excess solar generation on highly renewable grids.^36^ Our analysis indicates that midday charging between 10 a.m. and 3 p.m. will most effectively leverage peak solar generation. Shifting EV charging demand to the early afternoon can help utilize solar but still will not fully absorb the large daytime surplus in regions with abundant solar resources. In Texas, solar output can exceed demand by multiple gigawatts during peak solar availability.^37^ Overall, the coordination of EV charging during peak solar hours should be considered as one demand-side management strategy in a larger portfolio.

Vehicle-to-grid (V2G) technology enables EVs to both draw power from the grid and supply energy back when needed. This bidirectional capability allows EV owners to incorporate daytime charging, regardless of their initial state of charge (SoC). This could increase the value of midday charging. Through careful design, EVs may develop into assets for grid stability rather than liabilities associated with peak demand.

### Charging paradigms

Although this study presents theoretically sited charging stations rather than observed post-installation use, we compared the projected charging profiles with observed charging behavior reported in prior work. This comparison evaluates the core assumption that potential charging demand would broadly track visitation patterns. The projected charging profiles by POI category generally align with observed charging demand for categories that overlap with the existing infrastructure.^38^^,^^39^

To enable comparison, candidate sites were mapped to the previously defined^40^ categories used in Borlaug et al.,^38^ and categories without clear correspondence to existing charging-site classifications were excluded from this comparison. Notably, some candidate sites identified in this study are underrepresented or absent in existing charging networks. While these new categories may identify opportunities for new destination charging, we are unable to assess whether charging projections are reasonable for such sites.

**Figure 6:**
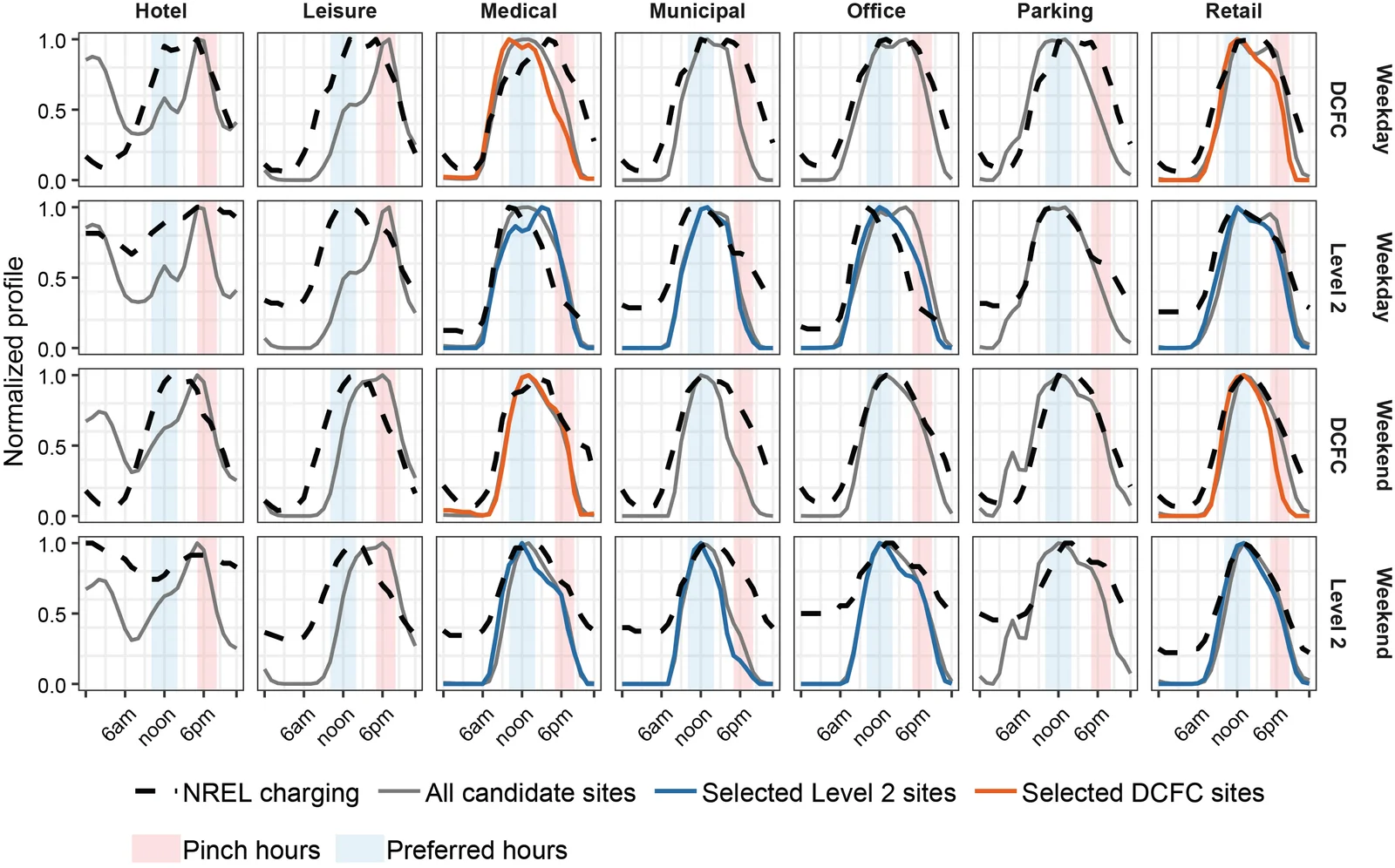
Comparison of visitation-derived profiles to observed charging behavior by category, charger type, and day type Each plot shows the normalized electricity demand from observed charging data (dashed lines) and the normalized visitation intensity for candidate sites (solid lines). For categories in which charger infrastructure is suggested, visitation profiles for the selected subset of sites are shown. Categories without proposed sites include only all-site visitation and observed charging demand. Correlations between observed charging profiles and visitation-derived profiles are reported in the supplemental information. See Figure S1 and Tables S1–S5.

Across most categories, the normalized visitation profiles closely match the observed charging-demand patterns (median correlation 0.92) as shown in Figure 6. However, discrepancies are present in categories with limited sites in the visitation dataset used here. For example, only 6 municipal and 17 leisure sites were represented, which may affect the comparability of the estimated visitation profiles. Full quantitative comparison metrics are provided in the supplemental information.

The results also indicate that the site selection process identifies a subset of the candidate sites with different behavior. For example, weekday office visitation at all sites has higher visitation later in the day, but for the subset selected for chargers, visitation declines earlier. A similar pattern is observed for retail locations, where all candidate sites exhibit higher pinch hour visitation than the selected subset of sites. These differences suggest that the selection framework is not only identifying high-traffic locations but also prioritizing sites with temporal profiles better aligned with desired charging. Thus, the contribution of this framework is not simply estimating where EV charging demand may occur but identifying where public charging could be sited to encourage demand during beneficial hours.

### Limitations of the study

This analysis utilized location-based mobility data from Spectus to determine which POIs are already popular at the preferred times. The data are collected from smartphone applications whose users have authorized high-resolution data collection and are then anonymized for privacy. The precise location data source does not represent the entire population, but prior studies^41^^,^^42^ have shown that the data correlate well with known population locations.

The projected load calculations here are based on optimistic assumptions to show the potential impact, not necessarily the most likely impact. Those assumptions include that users will adopt destination charging when available and convenient, and that in the near future, the general population will be representative of EV drivers as vehicles become more accessible. In reality, other limitations may slow behavior shifts, for example, other reasons for parking or charging preference, or a lack of awareness of public charging. Reliability of the charging stations themselves has also been identified as an issue affecting increased public charging.^43^

This study should be interpreted as a siting framework. The analysis does not include distribution grid constraints, voltage stability, grid optimization, or station-level control. It also does not optimize the exact configuration of chargers. Instead, the framework identifies locations where existing visitation patterns suggest that public charging could be more likely to occur during preferred hours and less likely during pinch hours. To support evaluation and adaptation of this method, we provide the code used in the supplemental information and report data sources in the data availability statement.

### Future work

The proposed charging paradigm is designed to address known grid challenges described in prior work^44^^,^^45^^,^^46^^,^^47^ but does not explicitly evaluate grid-level effects such as ramping or stability. Prior work has highlighted that the timing, magnitude, and location of EV charging are critical determinants of grid impacts. Poorly sited charging loads contribute to increased peak demand and degradation of voltage stability and reliability. Relative to studies that optimize charging operations after station locations are known,^11^^,^^15^^,^^19^ this work contributes an upstream siting approach that incorporates temporal grid conditions and observed urban activity patterns before infrastructure is deployed. Future work should couple the candidate locations identified here with distribution-grid models, formal optimization, sensitivity analysis, and post-deployment charging data to evaluate reliability, cost, and realized load shifting.

Ultimately, if loads are effectively managed over the course of a day, utilities can more efficiently utilize their generation infrastructure. However, substations, distribution lines, and other costly infrastructure upgrades may still be required if the spatial variation between distributed loads and generation is not considered.^48^ One way this can be achieved is through co-location of distributed solar with the EVCS combined with solar-based location selection. Future work could incorporate subgrid boundaries to evaluate EVCS placement with respect to the local-scale supply, demand, and distribution constraints.

The methodology for identifying POIs here is replicable in many other locations. By relying on widely available datasets, the procedure in Figure 1 can be replicated to identify candidate charging locations in any urban area.

New instances of LFLs are emerging through the use of artificial intelligence and cryptocurrency mining.^49^^,^^50^ Data centers can enter voluntary demand response agreements^50^ but otherwise risk exacerbating peak demands.^51^ Texas legislators have introduced a measure (16 TAC §25.114^52^) to mandate registration and participation in demand response, to limit load expansions and increase transparency of the curtailed load payments.^53^ This further highlights the need for demand-side management throughout the grid system.

Rising living standards and climate change are driving surging air conditioning demand, while expanding computational loads further increase demand. As these demands grow, maintaining grid stability will depend on harmonizing unique usage patterns with VRE generation. If loads are effectively managed, utilities can reduce the necessity for costly infrastructure upgrades. As peak loads and sudden increases in demand typically require higher emitting generation, emissions of both criteria air pollutants and greenhouse gases (GHGs) can also be reduced.^54^ By treating EVCSs as both transportation infrastructure and a demand-side management tool, cities may broaden access to EVs and demand-side management opportunities while improving renewable energy utilization.

## Resource availability

### Lead contact

Requests for further information and resources should be directed to and will be fulfilled by the lead contact, Kristen E. Brown (kristen.brown2@utsa.edu).

### Materials availability

This study did not generate new unique reagents.

### Data and code availability

• This paper analyzes existing, publicly available data for energy demand, projected curtailment, and existing charging infrastructure, accessible at https://www.ercot.com/gridinfo/load/forecast, https://scenarioviewer.nrel.gov, and https://afdc.energy.gov/data_download, respectively.
• The mobility data reported in this study cannot be deposited in a public repository due to licensing restrictions. To request access, contact Dewey Data for SafeGraph at deweydata.io and Cuebiq for Spectus at cuebiq.com/demo.
• All original code is available at https://github.com/Kris10Brown/Renewable_Aligned_EV_siting.
• Any additional information required to reanalyze the data reported in this paper is available from the lead contact upon request.

## Acknowledgments

This project was funded by monies provided by CPS Energy through an agreement with The University of Texas at San Antonio.

## Author contributions

Conceptualization, K.E.B.; data curation, S.A.M., N.W., and E.A.L.O.; formal analysis, N.W., S.A.M., E.A.L.O., and K.E.B.; funding acquisition, K.E.B.; project administration, K.E.B.; software, N.W. and S.A.M.; visualization, K.E.B.; writing – original draft, N.W.; writing – review and editing, K.E.B.

## Declaration of interests

The authors declare no competing interests.

## STAR★Methods

### Key resources table

**Table:** 

REAGENT or RESOURCE	SOURCE	IDENTIFIER
**Deposited data**		

Forecasted load data	ERCOT’s publicly available grid information database	https://www.ercot.com/gridinfo/load/forecast
Energy system analysis datasets Cambium 2024 mid-case scenario	National Laboratory of the Rockies (NLR)	https://scenarioviewer.nlr.gov/
SafeGraph relative occupancy	Dewey Data	deweydata.io
geolocation data	Spectus	cuebiq.com/demo
existing charging infrastructure	Department of Energy	https://afdc.energy.gov/data_download

**Software and algorithms**		

Original Code	Original code	https://github.com/Kris10Brown/Renewable_Aligned_EV_siting

### Method details

#### Electricity projections

Forecasted load data were obtained from ERCOT’s publicly available grid information database.^55^ The forecasted net, base, large flexible, and EV loads, along with distributed PV generation for 2025 and 2030 are available for every hour of the year for both the entire ERCOT region and a subset of the grid serving central Texas, including San Antonio and Austin. Energy system analysis datasets for modeling electricity generation in ERCOT were obtained from the National Renewable Energy Laboratory (NREL) Cambium 2024 mid-case scenario.^32^

#### Spatial occupancy

Census block groups around the study area were evaluated for relative occupancy across the course of a day using Advan SafeGraph data from Dewey. This dataset provides data at the census block group level and counts all unique hourly visits. 31 block groups were identified as particularly popular during the preferred time. Individual addresses (potential POIs) were then assessed within these block groups using Spectus geolocation data to identify EVCS locations. The Spectus data provides the average, minimum, and maximum dwell time for each address in each month and the relative visitation popularity in each hour of a week during that month.

All potential POIs in the selected block groups were assessed and filtered for potential as a level 2 or DCFC site. Separate filtering criteria were used to identify DCFC and Level 2 charging sites. Minimum visitation count thresholds based on statistics within the data require that even within block groups that are popular at the preferred times, these are highly visited sites. The visitation characteristics are analyzed for each combination of a day of the week and month of the year, which should account for variation in busy seasons, days in which businesses are closed, and variation in activity across days of the week.

#### New load calculations

When estimating potential public charging demand, we use hourly visitation intensity profiles to represent the relative temporal distribution of potential charging activity. For each selected site, an hourly charging profile was constructed by scaling visitation intensity to the maximum available charging capacity at that site. We assumed that each Level 2 charger has 2 ports that draw 6.5 kW each while charging, so during maximum visitation each site can draw up to 13 kW at a time. For DCFC, we assume that each station has 4 ports that draw 250 kW each, enabling a load of up to 1,000 kW of load per site. These assumptions define an upper bound on site-level charging capacity. Hourly load profiles were generated for each site and aggregated across all selected locations to produce a system-level public charging profile.

### Quantification and statistical analysis

The “pinch” time is identified based on the degree to which the large flexible load (LFL) from the ERCOT adjusted long term load forecast deviates from the typical load. This demand response was determined by calculating F, the fraction of load in an hour compared to the typical load, as

$$F=\frac{L_{t}}{M}$$

Where *L*_*t*_ is the load in a given hour, and *M* is the median load across the year. Smaller values indicate demand response limiting demand at that time. The pinch times were identified as those in which the fraction (F) is less than 0.5, or the flexible loads are below 50% of their median value.

For comparison between previously observed charging load and the projected load, visitation-derived profiles were assigned to categories^40^ and compared with observed charging profiles. Categories without a clear correspondence were excluded. Profiles were normalized^38^^,^^39^ independently to their maximum hourly value and compared separately by charger type and day type. The comparison was quantified using mean absolute error () between the hourly values, difference in the category peak hour (difference between the hours of the day in which the highest demand occurs), and Pearson correlation coefficient between sets of curves. Metrics were calculated separately for each charger type, day type (weekday vs. weekend) and category. Statistics can be found in the supplemental information, including the number of sites evaluated in each category.

Agreement was quantified using Pearson’s correlation coefficient and mean absolute error (MAE) across the 24 hourly observations. The difference in peak timing was calculated as the difference between the hour in which the observed and projected charging profiles reached maximum. Peak hour *H* was defined for each profile (*obs* for observed and *proj* for the profile projected in the paper) as the hour of the day in which the highest charging draw occurs. And the peak difference (Δ) was defined as

$$\Delta =H_{obs}-H_{proj}$$

so that positive numbers indicate the visitation based projection peaks earlier than the observation. The number of POIs contributing to each visitation profile is also reported because estimates based on small categories may be less representative.
