# Supplementary material for: A framework for siting public EV charging during renewable-aligned hours
Source: iScience. 2026 Aug 13;29(9):117198. doi: 10.1016/j.isci.2026.117198 (PMC13495402; doi:10.1016/j.isci.2026.117198)
Supplement: Supplementary file 1 [file mmc1.pdf]

iScience, Volume *29*

Supplemental information

A framework for siting public EV charging during renewable-aligned hours

Nicolas Williams, Salomon A. Mendoza, Esteban A. Lopez Ochoa, and Kristen E. Brown

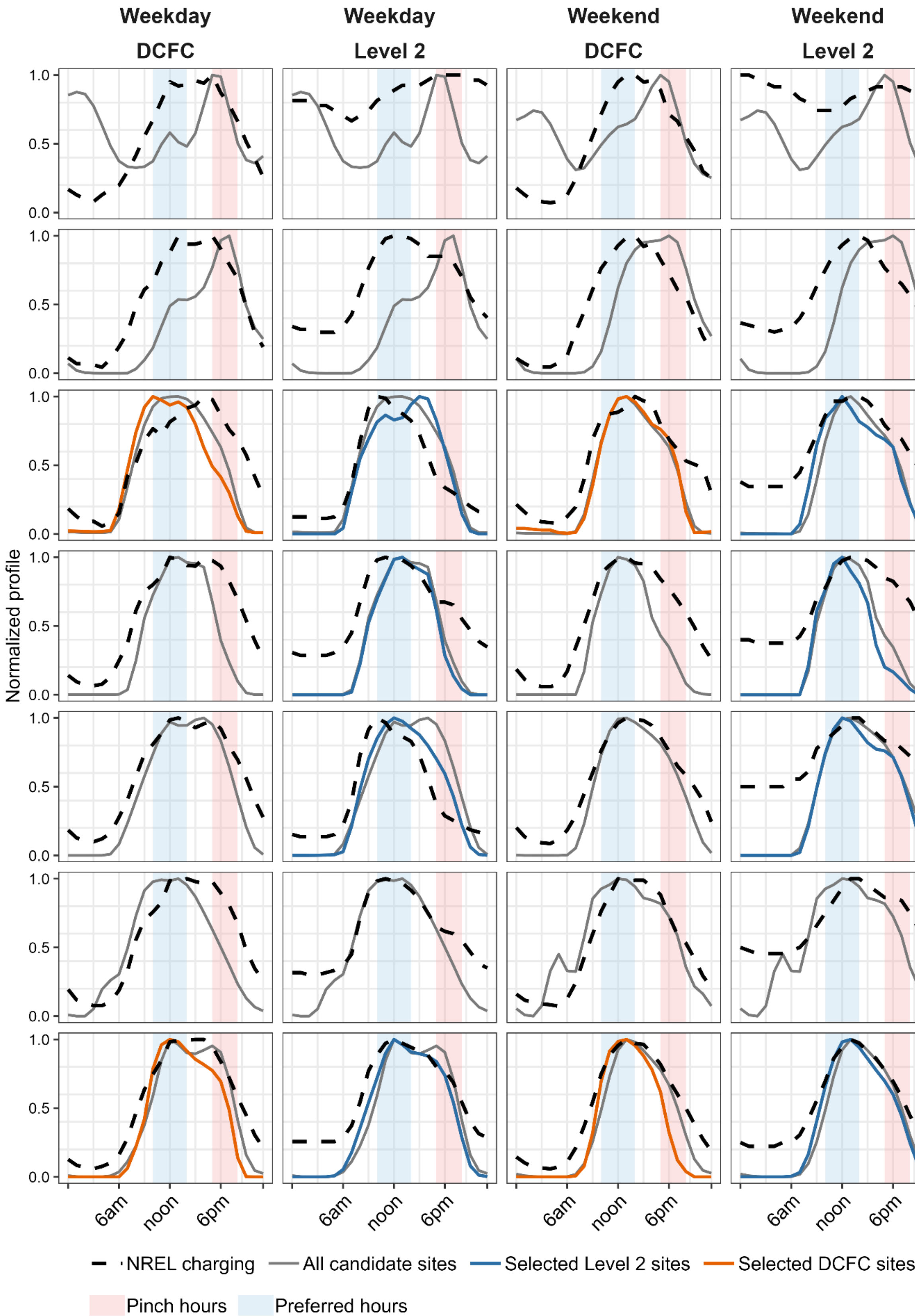

Figure S1 Expanded presentation of Figure 6, rearranged to show detail more easily.

Table S1. Quantitative comparison of visitation-derived and observed charging profiles.

Comparison	n	MAE	Peak difference mean (range)	Correlation mean (range)
**All visits v NREL DCFC**	14	0.183	-0.64 (-6 to +3)	0.806 (0.084-0.984)
**All visits v NREL L2**	14	0.239	-2.79 (-17 to +1)	0.830 (0.359-0.991)
**Suggested DCFC v NREL DCFC**	4	0.180	2.50 (0 to +6)	0.887 (0.749-0.946)
**Suggested L2 v NREL L2**	8	0.231	-0.75 (-5 to +1)	0.937 (0.834-0.996)

Table S2. All Candidate sites vs observed DCFC

Category	Day type	Sites in category	Correlation	MAE	Peak-hour difference
**Hotel**	Weekday	101	0.084	0.302	0
**Leisure**	Weekday	17	0.8	0.226	-6
**Medical**	Weekday	48	0.866	0.179	3
**Municipal**	Weekday	6	0.868	0.215	-1
**Office**	Weekday	350	0.973	0.136	-3
**Parking**	Weekday	1	0.826	0.192	1
**Retail**	Weekday	2613	0.976	0.115	3
**Hotel**	Weekend	101	0.404	0.26	-4
**Leisure**	Weekend	17	0.782	0.196	-4
**Medical**	Weekend	48	0.939	0.168	1
**Municipal**	Weekend	6	0.897	0.216	1
**Office**	Weekend	350	0.984	0.117	0
**Parking**	Weekend	1	0.908	0.12	0
**Retail**	Weekend	2613	0.974	0.121	0

Table S3. All candidate sites vs observed Level 2

Category	Day type	Sites in category	Correlation	MAE	Peak-hour difference
Hotel	Weekday	101	0.359	0.29	0
Leisure	Weekday	17	0.7	0.332	-7
Medical	Weekday	48	0.935	0.131	-3
Municipal	Weekday	6	0.933	0.262	-2
Office	Weekday	350	0.767	0.213	-6
Parking	Weekday	1	0.963	0.136	-2
Retail	Weekday	2613	0.957	0.187	0
Hotel	Weekend	101	0.506	0.289	-17
Leisure	Weekend	17	0.756	0.276	-4
Medical	Weekend	48	0.982	0.258	0
Municipal	Weekend	6	0.921	0.321	1
Office	Weekend	350	0.991	0.287	0
Parking	Weekend	1	0.859	0.21	1
Retail	Weekend	2613	0.987	0.156	0

Table S4. Selected DCFC sites vs observed DCFC

Category	Day type	Sites in category	Correlation	MAE	Peak-hour difference
Medical	Weekday	15	0.749	0.228	6
Retail	Weekday	2	0.946	0.162	3
Medical	Weekend	15	0.923	0.161	1
Retail	Weekend	2	0.931	0.168	0

Table S5. Selected Level 2 sites vs observed Level 2

Category	Day type	Sites in category	Correlation	MAE	Peak-hour difference
Medical	Weekday	4	0.883	0.162	-5
Municipal	Weekday	1	0.922	0.276	-2
Office	Weekday	15	0.903	0.158	-2
Retail	Weekday	84	0.989	0.169	0
Medical	Weekend	4	0.981	0.249	1
Municipal	Weekend	1	0.834	0.364	1
Office	Weekend	15	0.986	0.315	1
Retail	Weekend	84	0.996	0.157	0
